# Social Hair Plucking’s Associations with Other Behaviors and Its Health Considerations, in Captive Rhesus Macaques (*Macaca mulatta*)

**DOI:** 10.1080/10888705.2026.2692473

**Published:** 2026-06-30

**Authors:** Alexander J. Pritchard, Julia A. Salamango, Brenda McCowan

**Affiliations:** a National Biomedical Research Institute, University of California Davis, Davis, CA, USA; b Department of Population Health & Reproduction, School of Veterinary Medicine, University of California Davis, Davis, CA, USA; c Department of Anthropology, University of California, Santa Barbara, CA, USA

**Keywords:** Social hair pulling, abnormal behavior, stereotypy, social dominance, behavioral management, trichotillomania, barbering

## Abstract

Primate social hair plucking (SHP) has been posited to be prosocial or agonistic, though the health-associated concerns of SHP are less frequently explored. We studied SHP in seven mixed-sex groups of captive rhesus macaques (*Macaca mulatta*), at the National Biomedical Research Institute. We examined whether individual rates of SHP were associated with grooming, aggression, and socio-demographic characteristics. We also examined whether SHP rates were associated with alopecia, body condition, and hair cortisol concentrations. Rates of SHP production and reception were similar to grooming and aggression, yet exhibited directional rank flow similar to aggression with kin biases similar to those for grooming. SHP given was associated with shared kinship with a social hair plucker; putative evidence of social transmission. SHP received was associated with alopecia. SHP given was associated with hair cortisol concentrations in females, albeit with high uncertainty. SHP fits the description of an abnormal behavior, is defined by a function, shows characteristics upheld by prosocial and aggressive behaviors, and has health associations. Future research should develop a comparative framework of SHP across species to examine mechanisms and motivations.

## Introduction

Social hair pulling or plucking (SHP), where a social initiator removes hair from a recipient, is common in captive nonhuman primates across taxa, housing facilities, and social groups (Brand & Marchant, 2018; Heagerty et al., 2017; Laméris et al., 2021; Reinhardt et al., 1986). The underlying function and consequences of this behavior remain unclear in relation to social stability and animal welfare. Furthermore, the behavioral context governing its performance remains unresolved. SHP has been presented as a standalone behavior, but also in association with affiliative and agonistic behaviors (Brand & Marchant, 2019; Heagerty et al., 2017; Laméris et al., 2021; Reinhardt, 2005). Initiators show complex behavioral expression, including whether the hair is consumed. Recipients exhibit varied responses, from apparent tolerance or indifference (Heagerty et al., 2017; personal observations, AJP, JAS, 2022–2024) to irritancy or submission (Reinhardt, 2005; Reinhardt et al., 1986; personal observations, AJP, JAS, 2022–2024). This lack of cohesive clarity necessitates a deeper understanding of SHP.

Numerous lines of evidence suggest that SHP can be functionally similar to affiliative behaviors. In captive bonobos (*Pan paniscus*), Brand and Marchant (2019) reported an association between dyadic rates of grooming and SHP. Similarly, rates of SHP were predicted by Sociability personality scores from behavioral observations of bonobos across numerous zoos (Laméris et al., 2021). In rhesus macaques (*Macaca mulatta*), Reinhardt et al. (1986) noted that SHP could occur during grooming bouts. Heagerty et al. (2017), however, never observed grooming and SHP within the same behavioral bout, though they did not test whether grooming and SHP were associated.

SHP can occur in agonistic or explicitly aggressive contexts. SHP has been characterized in rhesus macaques as an agonistic-like behavior emerging from overcrowding (Reinhardt, 2005; Reinhardt et al., 1986). Higher-ranking individuals plucked in 96% of observed events and recipients exhibited aversive responses (Reinhardt et al., 1986). Heagerty et al. (2017) also reported a rank-bias in SHP, but with few fear-associated responses to SHP. Even so, concurrent aggression (within 4 min of SHP) was rare. Brand and Marchant (2019), however, found no association between rates of SHP and social dominance rank.

Thus, SHP has been described in various affiliative and agonistic social contexts. In settings of social isolation, self-directed hair plucking (self-plucking, hereafter) has been characterized as a stereotypy or abnormal behavior (Lutz et al., 2003; Polanco, 2021). In captivity, stereotypic behaviors are deviations from species-typical repertoires typified as repetitive and compulsive (Berkson, 1983; Mason, 2006), and can be indicative of housing or welfare concerns (Gottlieb et al., 2015; Lutz et al., 2003; Vandeleest et al., 2011). Though stereotypies could function as a stress coping strategy (Lutz & Baker, 2023). SHP and self-plucking have been conflated and might be anticipated to be causally linked given the similarities in form. Though high lifetime cumulative stress scores (Polanco, 2021) and urinary cortisol concentrations (Brand et al., 2016) have been associated with self-plucking, but not SHP. Such evidence weakens the link between self-plucking and SHP, as well as the rationale that SHP is a stereotypy.

Qualities of SHP parallel barbering behavior in captive rodents (i.e., *Mus musculus*; *Rattus norvegicus*). Barbering manifests as the compulsive removal of hair and whiskers from social others, resulting in alopecia (Garner, Weisker, et al., 2004). There is evidence of a female bias for initiating barbering. Dominant and subordinate mice barber, though the severity of barbering is greater “down” the hierarchy from dominant-to-submissive (Garner, Dufour, et al., 2004). Barbering is pronounced in overcrowded housing (Cooper et al., 2021; but see, Garner, Dufour, et al., 2004), as posited for SHP (Reinhardt, 2005; Reinhardt et al., 1986). Both barbering and SHP have been likened to trichotillomania in humans (Garner, Dufour, et al., 2004; Reinhardt et al., 1986). Trichotillomania, however, is typified as self-plucking with few studies describing SHP (trichotillomania-by-proxy). Trichotillomania-by-proxy has only been described among people exhibiting self-plucking (Beattie et al., 2009; De Sousa, 2015; Falkenstein & Haaga, 2016). Though more than half of surveyed adults who exhibit trichotillomania also self-reported performing trichotillomania-by-proxy, typically toward familiar recipients (Falkenstein & Haaga, 2016).

### Aims

We sought to clarify the performance, reception, and biological consequences of SHP. We used several years of data from captive rhesus macaques to address several aims: 1) Given contradictory evidence, what are the behavioral and demographic associations of SHP in our population? 2) Do the behavioral and demographic influences on SHP vary across contexts? 3) Do we find a relationship between SHP and health-associated measures among initiators or recipients?

### Behavioral and demographic associations with social hair plucking

Given that SHP coincides with prosocial behaviors in bonobos (Brand & Marchant, 2015, 2018, 2019; Laméris et al., 2021), we predicted that SHP rates would be associated with rates of grooming. Additionally, females (Brand & Marchant, 2018; Falkenstein & Haaga, 2016; Garner, Weisker, et al., 2004; Heagerty et al., 2017), as frequent groomers, would exhibit high rates of initiating or receiving SHP. We also predicted SHP would flow “down” the hierarchy (Heagerty et al., 2017; Reinhardt et al., 1986): lower ranking individuals would receive SHP from higher ranking individuals. Finally, given affiliative behavior can be biased toward kin (Kapsalis & Berman, 1996; Pritchard et al., 2025; Thierry, 2007), we predicted preferential SHP between kin relative to non-kin. This latter expectation, however, is complicated as social transmission might also occur among kin, thus we analytically separated kin that gave SHP from those that did not. Preferential exchange between kin that both practiced SHP could be explained by affiliative kin biases or social transmission, while SHP that flowed to kin that did not practice SHP would only support a kin bias. These predictions are important to resolve in a single study to reconcile inconsistencies across studies.

### Behavioral and demographic associations of social hair plucking in an agonistic context

Importantly, Reinhardt et al. (1986) emphasized that SHP can occur in distinct contexts. At our research site, we have observed SHP occurring when the receiving animal is in a submissive crouch or pinned by one or more aggressors that contribute with SHP (Personal Observations, JAS, AJP, 2022–2024). Thus, SHP can occur in an explicitly aggressive context, but this distinction is not clearly demarcated in the literature. Indeed, Heagerty et al. (2017) reported that SHP was uncommon in an aggressive context. A lack of formal clarity between studies as to context and nuanced behavioral differences across contexts could lead to jingle-jangle fallacies whereby distinct behaviors are described by the same term (jingle), or the same behavior has different descriptions (jangle) (Carter et al., 2013; Kelley, 1927; Thorndike, 1903; Uher, 2011).

Thus, we conducted an exploratory analysis to determine whether SHP in the context of explicit aggression or submission showed the same socio-behavioral associations as SHP. We term SHP occurring in an aggressive context as agonistic social hair plucking (AHP, hereafter) to maintain clarity when discussing this behavior and reserve SHP for non-aggressive contexts, hereafter. This analysis is important for determining whether AHP and SHP are distinct behaviors expressed in disparate contexts or are under similar motivating or governing processes (i.e., SHP *sensu lato*, hereafter). We examined whether rates of initiated SHP and AHP were associated across individuals, and whether any behavioral and demographic characteristics were similar between Aims 1 and 2.

### Health-associated measures and social hair plucking

Broadly, we hypothesized that SHP would coincide with three health-associated measures: alopecia, body condition, and hair cortisol.

We predicted that individuals receiving SHP would exhibit greater alopecia, relative to those who did not (Heagerty et al., 2017; Novak & Meyer, 2009). Though we note that alopecia could occur with other underlying conditions (Mundy et al., 1998; Novak & Meyer, 2009).

We examined body condition scores both as a nutritional- and health-proxy, whereby SHP could directly or indirectly reflect or alter nutritional intake or general health. Indeed, the consumption of hair, which often occurs among individuals who give SHP, could be indicative of nutritional imbalances (Borgna-Pignatti & Zanella, 2016; Heagerty et al., 2017). Among animals that received SHP, however, we might expect greater distress and chronic hair removal that both impede well-being. Thus, we predicted an association between rates of SHP and variation in body condition, likely manifesting via emaciation.

Finally, we examined the association between SHP and hair cortisol. Cortisol is a biomarker of allostatic load (Wingfield & Romero, 2015) which has been associated with psychosocial stress (Sapolsky, 2004, 2005). We emphasize cortisol’s function as a metabolic hormone (Wingfield & Romero, 2015) whereby high or low cortisol is not necessarily a marker of poor welfare (Richardson, 2024). Even so, hair cortisol has utility due to its association with physiological activation via the hypothalamic-pituitary-adrenal (HPA) axis (Sapolsky et al., 2000). Importantly, stereotypic behaviors have been associated with HPA-axis dysregulation (Novak et al., 2013). If we assume that SHP is a stereotypy (evidencing HPA-axis dysregulation, poor welfare, or stress coping) or a symptom of metabolic activity or disease, then we predicted higher rates of SHP would be associated with either higher or lower cortisol, relative to individuals not engaging in SHP. Either directional change might be inferred as HPA-axis dysregulation: high cortisol could indicate higher physiological activation (e.g., increased metabolic activity); low cortisol could indicate a blunted response due to, for example, physiological desensitization or exhaustion (e.g., Cyr & Romero, 2009). We did not ultimately examine this as a competing hypothesis due to unfavorable model comparisons (**S1**), but acknowledge that receiving SHP might also alter hair cortisol concentrations, either due to a physical or psychosocial burden.

## Methods

### Ethics statement

Our research met the standards of the International Guiding Principles for Biomedical Research Involving Animals. We received approval from the Institutional Animal Care and Use Committee of the University of California, Davis.

### Behavioral data collection

We collected data from seven outdoor-housed large mixed-sex social groups studied between 2012 and 2016, across nine study periods (Table 1). Two groups (A & D) were studied across two sampling periods. Subjects were adult and sub-adult (≥3 year old) rhesus macaques at the National Biomedical Research Institute (NBRI, formerly the California National Primate Research Center) at the University of California, Davis. The structure of data collection occurred as previously reported (Balasubramaniam et al., 2016, 2019; Pritchard et al., 2024): observers collected data for six h a day resulting in 24 h of observations spread across 4 days of each observation week. We used scan sampling for affiliative data and all occurrence event sampling for conflict data (Altmann, 1974). Per study period, we had a mean of 62.38 ± 18.99*sd* unique observation days for conflict and 60.13 ± 17.23*sd* unique days for affiliation.

Scan samples were conducted every 20 min. Observers would proceed systematically around the enclosure and record all dyadic occurrences of grooming and SHP. SHP was defined as a: “Jerking movement where one individual pulls out a few to a large chunk of another animal’s hair with fingers or teeth; often appears uncomfortable to the receiving animal.” SHP could be scored as tolerated or not tolerated. For each individual, we extracted directed counts of SHP and grooming.

Using event sampling, we recorded all observable instances of agonistic behavior: including dyadic aggression, redirects, interventions, and displacement submissions. We also scored any instances of AHP during event sampling. AHP was defined as: “Animal [initiator] pulls on skin or hair out of recipient despite recipient’s effort to turn or move away. Can be recorded in a context of ongoing aggression (in conjunction with pinning).” AHP could not be scored as tolerated. We used displacements to calculate ordinal social dominance rank using randomized Elo-ratings (Sánchez-Tójar et al., 2018), and scaled rank within group. We extracted counts of dyadic aggression (directed or redirected, as opposed to polyadic interventions) for aggression given and received, to use in models examining SHP and AHP. We excluded any instances of aggression that co-occurred with AHP to avoid associations driven by co-occurrence of data rather than independent rates of aggression. We normalized all counts of behavior as model predictors by the number of affiliation or conflict observation days to obtain a rate. For model response variables, we included observation days as an offset where appropriate.

### Temporal changes in social hair plucking

Although we had no explicit expectations regarding transmission, we explored data from the two groups with replicated study periods. We conducted this analysis *post hoc* to examine whether SHP behavior increased or decreased at the level of the group over a broad intervening time period (one or 2 years).

### Ratings of alopecia and body condition

Between 2013 and 2016, six of the groups’ (A [2014 only], B, D [2013 only], E, F, & G) subject animals (*N* = 507) were rated during their relevant observation periods from 1-to-5 on alopecia and general body condition. For alopecia, animals were rated as: “1” for “very good hair,” with regularly increasing 25% intervals for missing hair on subjects’ bodies such that a score of “5” would indicate 100% hair loss. For body condition, we set “3” as an “optimum,” relative to extreme scores of “1” for “very thin/emaciated” and “5” for “obese/grossly obese.” Subjects were rated approximately every week, though we reduced the frequency of body condition ratings in September, 2014, to fortnightly due to a lack of rapid changes in scores. We obtained 9,284 alopecia scores (average samples per subject = 18.31 M ± 4.33*sd*) and 6,582 body condition scores (average samples per subject = 13.01 M ± 6.16*sd*).

### Hair cortisol sampling and assays

Between 2013 and 2016, we collected hair samples from six study groups (A [2014 only], B, D [2013 only], E, F, & G). Hair cortisol was extracted and then assayed using enzyme immunoassays (Salimetrics, Carlsbad, CA, USA), as previously detailed (Vandeleest et al., 2019). Individual samples and assay plates passed a threshold of 15% for the interassay and intrassay coefficients of variation. We sampled three times within the same groups, except group “G” was sampled four times. Enclosures were repeatedly sampled in 5-to-10 week intervals (mean interval = 6.92 ± 1.93*sd* weeks). Sampling events included sedation of all adult subjects in the enclosure. We had 1,428 samples with hair cortisol data, but excluded 10 as outliers (>3*sd* from the mean) resulting in 1,418 samples from 521 individuals, across the six groups. We acquired a mean of 2.72 ± 0.84*sd* samples per animal subject.

### Model construction and selection

We ran all models with Bayesian regression using Stan (Bürkner, 2017) in the “R” programming language (R Core Team, 2022). Continuous predictors were scaled by two standard deviations (Gelman, 2008). Models were constructed as intercept-only models to confirm family fit, with the subsequent addition of random effects to confirm that their inclusion improved model fit. Model selection then proceeded with fixed effects of biological or environmental salience (e.g., sex, age). Variables that were collinear (e.g., rank and aggression) were selected between, to prevent multi-collinearity. Predictors of interest were retained in models. Model selection was performed using *loo_compare()* in *brms*. We selected models with clear separation from other candidate models (>2 * *se_diff* from the *elpd* of the best performing model) or that were more biologically appropriate based on our knowledge of the system. Model selection is detailed in the supplementary materials (**S1-S6**). Model fit was confirmed, across all models, by confirming Rhats were = 1.0 and that effective sample sizes were sufficiently high (e.g., >1000). This was true for all models. We confirmed model fit using posterior predictive checks and by examining pairs plots (**S7-S13**).

### Models for SHP given/received and AHP given

Models for rates of SHP given, SHP received, and AHP given were run using distinct models with negative binomial family distributions. SHP received was improved by the addition of a term for zero-inflation (**S3**). We included sex and age in all of the final models. Behavior models were computed at the individual level, whereby each individual received a single rate from each observation study period. As rank and aggression were highly correlated, we selected whichever effect improved model fit (**S2-S4**). For SHP given and AHP given, we constructed models using predictors of behaviors given. For SHP received, we used predictors of behaviors received. We had affiliation observation days as an offset in the SHP given and received models, and conflict observation days in the AHP given models. Offsets included all observation days. We had individual-level relocation data for the majority of the groups (A [2014 only], B, D [2013 only], E, F, & G) and incorporated those at the individual level. That is, any days individuals were not in their enclosure (e.g., due to hospitalizations) were subtracted from group-level observation days. We included random effects for group and individual subject; though only two groups were sampled twice (A & D) and the remaining subjects had a single data point (**S7**).

### Models for body condition and alopecia scores

We ran distinct cumulative logit family models with flexible thresholds to determine whether alopecia or body condition scores were predicted by: whether subjects received SHP during the observation period (dichotomous), and count of matrilineal kin that gave SHP during the observation period. We also included SHP given during the observation period (dichotomous) for the body condition model. While we had access to the aforementioned rates of SHP, we acknowledge the complexity whereby high past or unobserved incidences of SHP might also contribute to alopecia, and that high instances of alopecia could deter subsequent incidences of SHP behavior (due to a low availability of hair). Thus, we retained a simplified measure of SHP by including the presence of SHP as a dichotomous variable such that all individuals received a “0” until the date in the study during which they were observed engaging in SHP, at which point we recoded SHP received or given to “1” for the remaining sampling dates.

### Models for hair cortisol

Hair cortisol concentration was modeled using a lognormal family. We included temperature measurements from the UCD/NOAA Climate Station: USC00042294 (Atmospheric Science Program, Land Air and Water Resources, University of California Davis, 2025; Menne et al., 2012) to account for climatic variation that could alter cortisol concentrations. We constructed this model by introducing rates of given and received behaviors in a stepwise fashion and comparing model fit with the addition of each behavior, starting with SHP, aggression, and grooming. Behavioral rates were calculated for each subject during observation days that preceded the hair cortisol sampling event, and were not attributed to any other sampling event.

## Results

### Behavioral and demographic associations with social hair plucking

**Figure 1.**
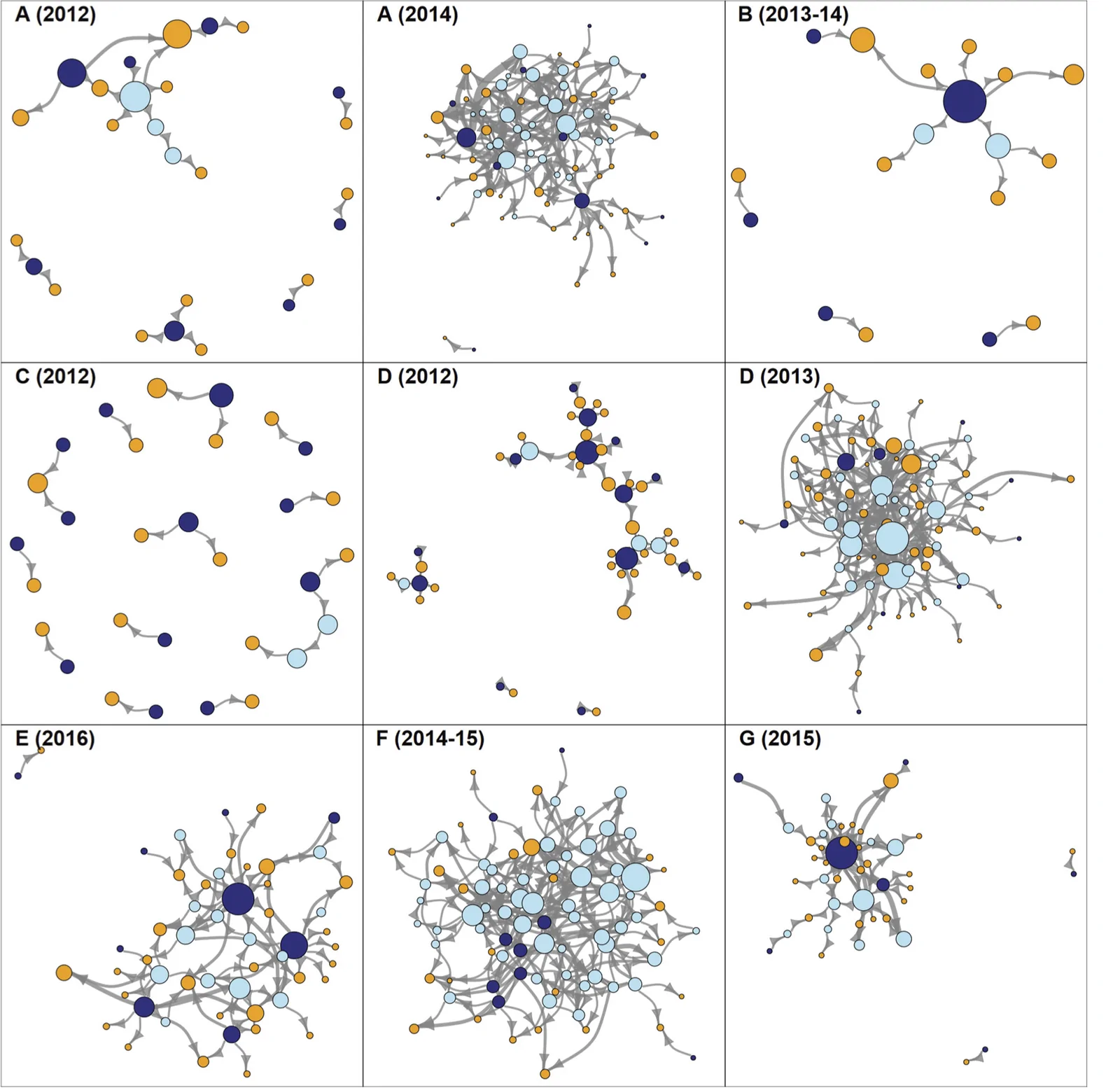
Social network graphs for SHP across the nine study periods. Each graph is labelled with the group code and year of observation (in parentheses). Nodes that only gave SHP are colored dark blue; nodes that only received SHP are colored orange; nodes that received and gave SHP are light blue. Edges are curved to emphasize direction of SHP, proceeding in a clockwise direction. Edges are weighted with a square root transformation to increase visibility of thin edges. Node size is equivalent to the total strength of SHP (given and received) with a square root transformation to increase visibility of small nodes. Graph dimensions are not scaled across groups.

Incidences of SHP varied dramatically by group and study year (Figure 1; Table 2). As grooming and SHP were scored dichotomously, we cannot provide estimates for the relative durations of SHP versus grooming. Contemporary observations, however, suggest that the performance of these behaviors can occur during the same bout (personal observations, AJP, JAS, 2022–2024). The majority of the 1078 observed SHP events were tolerated; only 11.13% SHP events were not tolerated among 24.47% of total dyads.

During conflict event sampling, we observed 919 instances of AHP. Just 40.81% of these observations occurred with initiator aggression, though 85.20% occurred with recipient submission or screaming. A minority (12.62%) did not co-occur with either aggression or submission, and did not involve the AHP initiator joining an ongoing conflict (i.e., an intervention). There are two alternative expectations why submission would not have been coded here: 1) Observers may have perceived turning away or moving away to be in response to the sensation of hair plucking rather than a submissive signal. 2) Observers could have inadvertently omitted these criteria.

As observers collected both protocols simultaneously, we examined whether AHP and SHP events were temporally concurrent. Between the SHP and AHP datasets, 23 events (0.8% of the total AHP and SHP events) were recorded from the same dyad within 10 min of each other and were likely not independent. Among the AHP behaviors, 17 instances (1.85%) were scored with present for grooming, social huddling, or grooming. Only 4 of these instances (00.44%) did not also include submission or occur as an intervention following a conflict event. We initially retained all instances of AHP for subsequent analyses, because we did not have *a priori* rationale that delineated a biological distinction between SHP and AHP beyond context of performance. After model construction, however, we re-ran our final model on data excluding the 126 AHP events (13.71%) without submission or aggression, with affiliation, or within ±10 min of a recorded SHP event.

### Social hair plucking given

The SHP given model included: sex, age, grooming given, aggression given, count of matrilineal kin (kin, hereafter) observed giving SHP as well as count of kin not SHP during the observation period; we included group and subject random effects. Rates of SHP given were associated with aggression given (estimate = 1.11, L-U 95% CI: 0.74 to 1.48), grooming given (estimate = 0.77, L-U 95% CI: 0.40 to 1.14), and the number of kin giving SHP (estimate = 1.12, L-U 95% CI: 0.70 to 1.53) (Table 3). The remaining matrilineal kin that did not give SHP was not associated with SHP given (estimate = −0.25, L-U 95% CI: −0.81 to 0.32). Males gave SHP at a lower incidence relative to females (male estimate = −1.04, L-U 95% CI: −1.56 to −0.52; reference = females). Age was not associated with SHP given (estimate = −0.05, L-U 95% CI: −0.49 to 0.39). Our model explained 74% of variation with random effects (conditional R^2^ = 0.74, error = 0.07, L-U 95% CI: 0.58 & 0.86) and 5% without (marginal R^2^ = 0.05, error = 0.05, L-U 95% CI: 0.005 & 0.196). If we reran the model without subject ID as a random effect, but retained the remaining model effects, then our model explained 26% of variation with random effects (conditional R^2^ = 0.26, error = 0.08, L-U 95% CI: 0.12 & 0.42), and 18% without (marginal R^2^ = 0.18, error = 0.11, L-U 95% CI: 0.03 & 0.44).

### Social hair plucking received

The SHP received final model included: sex, rank, age, grooming given, count of kin observed giving SHP as well as count of kin not giving SHP during the observation period; we included group and subject random effects. Rates of SHP received were associated with grooming received (estimate = 0.67, L-U 95% CI: 0.42 to 0.93), age (estimate = 0.46, L-U 95% CI: 0.23 to 0.70), the number of kin giving SHP (estimate = 0.61, L-U 95% CI: 0.37 to 0.86), and social dominance rank – whereby lower ranked individuals received higher rates of SHP (estimate = 0.79, L-U 95% CI: 0.55 to 1.04) (Table 3). The remaining matrilineal kin that did not give SHP was negatively associated with SHP received (estimate = −0.54, L-U 95% CI: −0.85 to −0.24). Males received SHP at a lower incidence relative to females (male estimate = −0.79, L-U 95% CI: −1.05 to −0.53; reference = females). Our model explained 41% of variation with random effects (conditional R^2^ = 0.41, error = 0.05, L-U 95% CI: 0.30 & 0.50) and 19% without (marginal R^2^ = 0.19, error = 0.10, L-U 95% CI: 0.04 & 0.40).

### Behavioral and demographic associations with agonistic hair plucking

The AHP given final model included: sex, age, SHP given, grooming given, aggression given, count of kin observed giving SHP as well as count of kin not giving SHP during the observation period; we included group and subject random effects. Rates of AHP given were associated with aggression given (estimate = 2.19, L-U 95% CI: 1.82 to 2.58) and SHP given (estimate = 3.56, L-U 95% CI: 2.34 to 4.78) (Table 4). Unlike SHP, grooming given did not predict AHP (estimate = 0.07, L-U 95% CI: −0.32 to 0.45). Subjects’ number of kin was not associated with AHP, irrespective of whether they performed SHP (estimate = 0.33, L-U 95% CI: −0.06 to 0.74) or not (estimate = 0.08, L-U 95% CI: −0.44 to 0.60). Males gave AHP at a lower incidence relative to females, albeit with high uncertainty (male estimate = −0.49, L-U 95% CI: −0.96 to −0.02; reference = females). Age was not associated with AHP given (estimate = −0.17, L-U 95% CI: −0.56 to 0.21). Our model explained 59% of variation with random effects (conditional R^2^ = 0.59, error = 0.06, L-U 95% CI: 0.46 & 0.69) and 27% without (marginal R^2^ = 0.27, error = 0.13, L-U 95% CI: 0.06 & 0.50). Outcomes were qualitatively and quantitatively similar when excluding 126 AHP events without submission or aggression, with affiliation, or within ±10 min of recorded SHP events (**S14**).

### Hierarchical flow and social hair plucking

We also examined the proportion of SHP, AHP, grooming, and aggression that flowed “down” the hierarchy (i.e., from high-to-low ranked individuals). We calculated a ratio of the number of interactions initiated by an individual that flowed down the hierarchy relative to the total numbers of interactions. We calculated similar ratios at the level of the dyad. We calculated these proportions among all individuals, among only matrilineal kin, and among non-kin. Note that the percentages of interacting kin-dyads, relative to the total number of dyads, were 26% for SHP, 11% for AHP, 7% for aggression, and 20% for grooming.

Interaction- and dyadic-level proportions were similar. SHP flowed down the hierarchy (65%) with a slightly stronger bias among non-kin (71%); hierarchical flow was not present among kin (50%). Dyadic-level proportions were similar: 68% across all dyads, 72% among non-kin, and 55% among kin. AHP flowed down the hierarchy (93%), with similar proportions among non-kin (95%) and, to a lesser extent, kin (78%). Dyadic-level proportions were similar: 93% across all dyads, 95% among non-kin, and 77% among kin. Aggressive interactions flowed down the hierarchy (82%), with a similar proportion among non-kin (83%), and, to a lesser extent, kin (74%). Dyadic-level proportions were similar: 74% across all dyads, 74% among non-kin, and 68% among kin. Finally, grooming interactions flowed up the hierarchy (41%), with an even lower proportion among non-kin (37%); hierarchical flow was not present among kin (51%). Dyadic-level proportions were similar: 42% across all dyads, 70% among non-kin, and 51% among kin. Across our groups, we did not find differences in the direction of rank flow for SHP among non-kin. Rank flow for SHP among kin, however, exhibited greater variation across the groups (six flowed up, two exhibited no flow, and one exhibited weak downward flow).

### Temporal changes in social hair plucking

The two replicated groups exhibited a marked increase in the percentage of adult animals that initiated SHP: increasing from 14.1% to 43.6% and from 17.0% to 38.2% in groups “A” and “D,” respectively. Within group “A”: of the 44 individuals that were observed giving SHP in 2014, 8 were observed giving SHP among the 31 present in 2012. Within group “D”: of the 39 individuals that were observed giving SHP in 2013, 14 were observed giving SHP among the 36 present in 2012. For subjects that were observed SHP in the second period, but not the first: 21.74% and 72.73% had kin that were observed giving SHP in the first observation period for groups “A” and “D,” respectively.

### Health-associated measures and social hair plucking

#### Alopecia and social hair plucking

The full alopecia model included age, sex with reproductive status (male, female-pregnant, female-not pregnant), SHP received, dominance rank, and count of kin observed giving SHP, with month as a cyclic spline; random effects included subject and group on the intercept, with group and observer on the discrimination parameters. High alopecia ratings were associated with SHP received (estimate = 0.55, L-U 95% CI: 0.30 to 0.89) and count of SHP kin (estimate = 0.29, L-U 95% CI: 0.03 to 0.58) (Table 5). Males received similar scores as the reference group (male estimate = 0.06, L-U 95% CI: −0.54 to 0.65; reference = cycling or lactating females). Pregnant females, however, had lower scores relative to the reference group (pregnant female estimate = −0.73, L-U 95% CI: −1.15 to −0.42; reference = cycling or lactating females). Rank was associated with differences in alopecia, such that lower ranked individuals had higher scores (estimate = 0.64, L-U 95% CI: 0.32 to 1.06). Age was not associated with differences in alopecia scores (estimate = 0.09, L-U 95% CI: −0.05 to 0.24). Based on Bayesian pseudo-R^2^, which assumes a continuous latent variable for ordinal models (Gelman et al., 2019; McKelvey & Zavoina, 1975), our model explained 79% of variation with random effects (conditional R^2^ = 0.79, error = 0.06, L-U 95% CI: 0.66 & 0.89), and 40% without (marginal R^2^ = 0.40, error = 0.09, L-U 95% CI: 0.24 & 0.58).

### Body condition and social hair plucking

The full body condition model included age, sex with reproductive status, SHP given and received, dominance rank, and count of kin observed giving SHP, with month as a cyclic spline; random effects included subject and group on the intercept, with group and observer on the discrimination parameters. Body condition was not associated with SHP given (estimate = −0.08, L-U 95% CI: −0.28 to 0.11) or received (estimate = −0.01, L-U 95% CI: −0.17 to 0.16), or count of kin that gave SHP (estimate = −0.30, L-U 95% CI: −1.00 to 0.34) (Table 5). Rather, males had lower scores relative to the reference group (male estimate = −1.84, L-U 95% CI: −2.73 to −1.10; reference = cycling or lactating females), indicative of lower body condition. Pregnant females had higher scores relative to the reference group (pregnant female estimate = 0.28, L-U 95% CI: 0.07 to 0.55; reference = cycling or lactating females), indicative of higher body condition. Higher ranked individuals had higher scores (estimate = −0.39, L-U 95% CI: −0.75 to −0.09), relative to lower ranked individuals. Generally, older individuals had higher scores, relative to younger individuals (estimate = 2.36, L-U 95% CI: 1.48 to 3.40; reference = cycling or lactating females). Our model explained 79% of variation with random effects (conditional R^2^ = 0.79, error = 0.07, L-U 95% CI: 0.62 & 0.89) and 49% without (marginal R^2^ = 0.49, error = 0.09, L-U 95% CI: 0.29 & 0.65).

### Hair cortisol and social hair plucking

The full hair cortisol model included: sex, age, SHP given, grooming given as well as received, aggression given, temperature (average maximum temperature, prior to sampling), with an interaction between SHP given and sex. We included group and subject identifiers as random effects. SHP given was very weakly-to-negligibly positively associated with hair cortisol (estimate = 0.04, L-U 95% CI: 0.00 to 0.07) (Table 6). We included an interaction term, as the previous models reported here emphasized a sex difference in SHP given, but the interaction did not credibly contribute and had wide credible intervals relative to other model terms – indicative of high estimate uncertainty (estimate = −0.10, L-U 95% CI: −0.21 to 0.00). *Post hoc* comparisons of the interaction showed very weak support for the SHP influencing cortisol in females (SHP trend = 0.04, lower HPD = 0.01, upper HPD = 0.07), while males were not credibly different from zero (SHP trend = −0.07, lower HPD = −0.17, upper HPD = 0.03). Aggression given (estimate = −0.04, L-U 95% CI: −0.07 to −0.01) was weakly associated with hair cortisol concentrations, such that higher rates of aggression given were associated with lower cortisol, relative to lower rates. Maximum temperature (estimate = −0.23, L-U 95% CI: −0.26 to −0.21) was negatively associated with hair cortisol concentrations, such that warmer maximums were associated with lower cortisol, relative to cooler maximums. Sex (estimate = −0.02, L-U 95% CI: −0.06 to 0.03), aggression received (estimate = −0.03, L-U 95% CI: −0.06 to 0.01), and age (estimate = 0.03, L-U 95% CI: −0.01 to 0.06) were not associated with hair cortisol concentrations. Our model explained 48% of variation with random effects (conditional R^2^ = 0.48, error = 0.02, L-U 95% CI: 0.45 & 0.52) and 15% without (marginal R^2^ = 0.15, error = 0.02, L-U 95% CI: 0.12 & 0.19).

## Discussion

All seven of our study groups expressed SHP. SHP showed concordance with prosocial and agonistic social behaviors, but with nuanced distinctions. We uniquely characterized instances of SHP in an agonistic context (AHP), which exhibited concordance with the propensity to give SHP – yet was defined by a lack of tolerance. Alopecia was associated with the prior occurrence of SHP received. We found very weak or equivocal evidence of an association between SHP and hair cortisol, in relation to the potential for health outcomes. Here, we explore these associations, reexamine the putative function of SHP, and provide a coarse exploration of potential social transmission.

### Behavioral and demographic associations with social hair plucking

We expected that SHP would coincide with the performance of prosocial behaviors (Brand & Marchant, 2015, 2018, 2019; Laméris et al., 2021; Reinhardt et al., 1986; but see; Heagerty et al., 2017). As predicted, high rates of grooming were associated with the incidence of SHP – a finding, to our knowledge, yet to be formally demonstrated in rhesus macaques. Similarly, females both gave and received SHP at higher rates than males, in line with species-specific patterns of grooming (Kulik et al., 2015). We also found that higher rates of aggression were associated with SHP.

Importantly, SHP exhibited nuanced associations with social dominance and kinship. In contrast to grooming, SHP flowed “down” the hierarchy whereby lower ranked individuals were frequent recipients, relative to higher ranked individuals. Among kin, however, we did not find directional dominance rank biases; more similar to grooming (Matheson & Bernstein, 2000; Sade, 1972). SHP could be a privileged behavior among dominant individuals to which subordinates acquiesce, and/or SHP could be a dominance signaling behavior.

### Behavioral and demographic associations with agonistic hair plucking

SHP and AHP are unlikely to be completely distinct behaviors due to their similarity in form and because our recorded rates of AHP given were associated with rates of SHP given. Stated simply, SHP (*sensu lato*) likely subsumes AHP. In contrast with our findings for SHP, however, rates of AHP given were not associated with rates of grooming nor with the number of their kin that were observed SHP. Similarly, Heagerty et al. (2017) noted that SHP was uncommonly observed in aggressive contexts. AHP was defined by an aggressive context and, unlike SHP, was not tolerated by definition. Finally, rank biases for AHP were more similar to those found for overt aggression, especially among kin, relative to SHP. Thus, contextual variation was a persistent effect in our data. Future research on SHP (*sensu lato*) should be explicit about the context or “tone” of interactions. Whether AHP and SHP can be pooled would be contingent on the question.

### Health-associated measures and social hair plucking

The positive association between SHP received and alopecia is logical and expected (Heagerty et al., 2017; Novak & Meyer, 2009). Higher alopecia scores were also positively associated with the count of kin who were observed SHP, which suggests that SHP bouts among kin might have been more frequent than those we observed. Additionally, lower ranked individuals had higher scores of alopecia, relative to high ranked individuals. This finding is relevant given lower ranking animals were frequent SHP recipients. Although we note that rank effects beyond SHP could drive this dynamic. Body condition was not associated with SHP, weakening the hypothesis that SHP emerges from nutritional or health deficits – at least for deficits resulting in observable differences in body condition.

Hair cortisol concentrations and SHP given were equivocally associated. The certainty of this estimate was weak and the effect was limited to females. Though we did not formally test SHP received, its model inclusion was not supported via model comparisons (**S1; S6**). As aggression proceeds down the hierarchy (Hobson et al., 2021), our aggression findings are congruent with rank-cortisol associations (Sapolsky, 2005). Though rank-cortisol associations are not ubiquitous (Cavigelli & Caruso, 2015; Hoffman et al., 2010). Despite aggression and SHP exhibiting a similar hierarchical flow, we found an opposite directional association: SHP given was positively associated to hair cortisol. We cannot resolve the precise mechanism behind this association, but nominate it as evidence for potential HPA-axis dysregulation. That is, the small estimate warrants future research, yet our work suggests that SHP could reflect health-associated distinctions. Indeed, in 2024, one of this study’s SHP subjects had attained alpha male status and was hospitalized with a trichobezoar (hairball). Prior to hospitalization, he had been observed giving SHP with hair consumption (personal observation, JAS, 2024). His extended removal for medical treatment and recovery was disruptive to group stability. Thus, the health-related concerns of SHP are not trivial and have welfare- and treatment-related costs; including the welfare burden of social isolation as a treatment (Novak & Meyer, 2009).

### SHP across groups, evidence of social transmission?

The three groups with the highest rates of SHP (A [2014], D [2013], & F) were not readily distinguishable from other groups (Table 1 ; Table 2). Prior research indicated that SHP may result from overcrowding or be reduced during birthing seasons (Reinhardt et al., 1986). Contrary to these previous assertions, SHP and AHP could not be attributed to intergroup variation in density or observation season. Two of the three groups with the highest rates of SHP (A [2014] & F) did have the highest densities, but the second most prevalent SHP group (D [2013]) had the third lowest density. Two of the three groups with the highest rates of SHP (A [2014] & F) were primarily observed in the birthing season, but prior work suggested SHP is expected to be at its lowest during this time due to reduced female social activity (Reinhardt et al., 1986). We note that group “A” (2012) was the only replicate where the majority of data was not collected during a breeding or birthing season.

Two groups (A & D) with the highest rates of SHP (*sensu lato*) were replicates; though, this was not by design. Between the study periods, both groups increased in the prevalence of SHP (*sensu lato*). For one group, the majority of newly observed individuals giving SHP in the second period had kin that were previously seen SHP in the first period. We posit that social transmission of SHP is likely, as asserted in bonobos (Brand & Marchant, 2019; Laméris et al., 2021). We substantiate this with our other reported findings: a higher incidence of kin-dyads among SHP pairs compared to grooming and aggression, a reduced rank bias of SHP among kin relative to non-kin, and an association between the count of kin giving SHP and rates of SHP given.

### Comparative parallels

It is most parsimonious to view SHP, barbering, and trichotillomania as similar in root cause and function, given the prevalence of these behaviors among females (Brand & Marchant, 2018; Falkenstein & Haaga, 2016, Garner, Weisker, et al., 2004; Heagerty et al., 2017; but see; Grant et al., 2020) and their associations with grooming or its context (Brand & Marchant, 2015, 2018, 2019; Garner, Dufour, et al., 2004; Laméris et al., 2021; Militzer & Wecker, 1986; Stein et al., 1999). Similar to barbering in mice (Garner, Dufour, et al., 2004), SHP was less likely to flow up the hierarchy, but such occurrences did exist. AHP, though, occurred almost exclusively down the hierarchy.

Periods of high-intensity aggression could evoke anxious arousal states that motivate AHP. In humans, the affective experience of trichotillomania is described as anger and sadness that steadily increased during and after plucking, relative to descriptions before hair plucking (Diefenbach et al., 2002). Similarly, trichotillomania often occurs with other “nervous habits” (Stein et al., 1999) or coincides with a state of nervousness as a precursor (Azrin et al., 1980). Progressive examination of arousal states before, during, and after SHP (*sensu lato*) would be of great utility in resolving congruence with the experience of trichotillomania.

We emphasize that, among macaques, SHP and self-plucking need not share a root cause (Polanco, 2021). The same may be true for trichotillomania or trichotillomania-by-proxy. More details are needed about the latter in humans as reporting of trichotillomania-by-proxy is likely impeded due to experienced shame and stigmatization (Beattie et al., 2009; Falkenstein & Haaga, 2016). In reference to barbering, Garner, Dufour, et al. (2004) emphasized its classification as an abnormal behavior, *not* a stereotypy – due to its goal-oriented focus and complex motor patterns. We advocate for the same nuance to SHP.

Resolving comparative perspectives and the expression of SHP is an important next step for devising management strategies, across taxa and sites. We should consider wild populations, as we encountered at least one anecdote of SHP in the wild. Multiple females, and frequently the alpha female, were observed SHP during a 2017–18 field study of rhesus macaques at Shimla, India (personal communication, B.A. Chakraborty, April 23 2024). Though anecdotal, such evidence substantiates the possibility that SHP can emerge in wild settings and should not be disregarded. To our knowledge, the only management strategy for SHP in captive macaques is the relocation and isolation of animals (Novak & Meyer, 2009). Trichotillomania is managed through habit training (Azrin et al., 1980; Mouton & Stanley, 1996): replacing the behavior with an alternative occupation of the subject’s hands (Duke et al., 2010). General enrichment, for instance, could promote manual activities that reduce the occurrence of SHP, as evidenced in mice (Bechard et al., 2011). Falkenstein and Haaga (2016), however, suggested that habit training may be an insufficient treatment for trichotillomania-by-proxy and advocated for understanding the social dynamics of the behavior. Finally, both trichotillomania and barbering have shown responsiveness to pharmaceutical interventions (Melo et al., 2022; Vieira et al., 2017). A combined approach of management strategies should be guided by deeper understandings of the individual- and group-level dynamics of SHP.

## Conclusion

SHP presents a compelling behavior that exhibits rank biases comparable to agonistic behaviors, but kin biases comparable to grooming behaviors. This behavior exhibits versatility and plasticity in its expression across multiple contexts, even though the behavior is similar in form. Future higher resolution behavioral analyses could reveal proximate motivations for initiating SHP, as opposed to grooming or agonism. Nuanced recording of recipients’ responses may help disentangle the varied functions of SHP as a complex, albeit abnormal, social behavior. Finally, there are clear parallels between barbering, social hair plucking, and trichotillomania (Reinhardt, 2005). Thus, we advocate for research to synthesize the study of these behaviors in a comparative framework, rather than through a piecemeal process. Future work should explore if there are similar motivational or affective aspects between these behaviors, and introduce health-associated biomarkers, as we have done here, to aid interpretation.

## Supplementary Material

Supp 1

Supplemental data for this article can be accessed online at https://doi.org/10.1080/10888705.2026.2692473

## Figures and Tables

**Table 1.** Group demographics and study details.

	Groups								
	
	A		B	C	D		E	F	G

Years Since									
Group Formation	6	8	9	5	21	22	14	11	8
Study Months, Year	May-Aug., 2012	Mar.-Jul., 2014	Sep.-Jan., 2013–14	Sep.-Dec., 2012	Feb.-May, 2012	Mar.-Jul., 2013	Mar.-Jul., 2016	Sep.-Jan., 2014–15	Mar.-Sep., 2015
Male : Female Counts (≥3yo.)	25 M: 59 F	34 M: 67 F	16 M: 39 F	31 M: 58 F	26 M: 75 F	27 M: 75 F	24 M: 57 F	28 M: 68 F	20 M: 54 F
Male : Female Counts	72 M: 97 F	91 M: 113 F	43 M: 82 F	74 M: 94 F	43 M: 104 F	36 M: 96 F	67 M: 92 F	79 M: 106 F	37 M: 70 F
Total Group Member Count	169	204	125	168	147	132	159	185	107
Sex Ratio (M : F ≥ 3yo.)	0.42	0.51	0.41	0.53	0.35	0.36	0.42	0.41	0.37

**Table 2.** Percentage of subjects exhibiting SHP and AHP. Rates of SHP, AHP, aggression, and grooming. Rates were normalized by number of affiliation or conflict observation days.

		Groups								
	
		A		B	C	D		E	F	G

Subjects Exhibiting	Give	14.1%	43.6%	12.7%	17.2%	17.0%	38.2%	28.4%	63.5%	27.4%
SHP (%)	Receive	23.1%	67.3%	23.6%	19.5%	34.0%	72.5%	54.3%	80.2%	52.1%
SHP Rates	Give	0.008 M ± 0.025	0.040 M ± 0.095	0.005 M ± 0.020	0.005 M ± 0.012	0.011 M± 0.032	0.052 M± 0.152	0.021 M± 0.062	0.057 M ± 0.093	0.012 M ±0.051
(Mean ±*sd*)	Receive	0.008 M± 0.019	0.039 M± 0.051	0.005 M± 0.010	0.005 M± 0.010	0.011 M ±0.018	0.054 M ± 0.075	0.022 M± 0.030	0.059 M ± 0.073	0.012 M±0.019
Subjects Exhibiting	Give	7.7%	38.6%	14.5%	16.1%	16.0%	34.3%	30.9%	34.4%	27.4%
AHP (%)	Receive	17.9%	57.4%	14.5%	12.6%	30.0%	79.4%	30.9%	27.1%	52.1%
AHP Rates	Give	0.005 M ± 0.025	0.035 M± 0.127	0.004 M ± 0.012	0.005 M± 0.016	0.011 M ± 0.048	0.050 M ± 0.134	0.012 M ± 0.029	0.017 M ± 0.034	0.016 M± 0.057
(Mean ±*sd*)	Receive	0.005 M ± 0.012	0.031 M± 0.078	0.003 M ± 0.008	0.006 M ± 0.028	0.011 M± 0.021	0.046 M ± 0.049	0.012 M ± 0.030	0.021 M± 0.045	0.017 M± 0.030
Aggression Rates	Give	1.32 M ± 1.10	2.11 M ± 1.52	2.29 M ± 1.68	2.17 M± 1.47	2.06 M ± 1.61	2.62 M ± 1.45	2.23 M± 1.25	2.95 M ± 1.83	2.80 M ± 1.39
(Mean ±*sd*)	Receive	1.32 M± 0.636	2.07 M ± 1.03	2.23 M± 0.952	2.17 M± 1.32	2.06 M± 1.33	2.60 M ± 1.58	2.26 M ± 1.42	3.02 M ± 1.58	2.79 M ± 1.56
Grooming Rates	Give	0.70 M ± 0.35	0.96 M ± 0.39	1.02 M ± 0.58	0.98 M ± 0.50	0.86 M ± 0.47	1.11 M ± 0.56	0.84 M ± 0.37	1.21 M ± 0.65	1.03 M ± 0.56
(Mean ±*sd*)	Receive	0.70 M ± 0.46	0.96 M ± 0.63	1.03 M ± 0.61	0.98 M ± 0.60	0.86 M ± 0.55	1.12 M ± 0.54	0.84 M ± 0.54	1.24 M ± 0.68	1.03 M ± 0.66

**Table 3.** Models’ estimates for rates of SHP given (top) and received (bottom).

SHP Given							

		Est. Error	Credible Intervals				
		
	Est.		l-95%	u-95%	Rhat	Bulk_ESS	Tail_ESS

	**Group-Level Effects:**						
~Group (Levels = 7) sd(Intercept)	0.85	0.39	0.35	1.87	1	5865	7020
~ID (Levels = 627) sd(Intercept)	1.73	0.13	1.50	1.99	1	3200	5605
	**Population-Level Effects:**						
Intercept	−5.51	0.39	−6.26	−4.69	1	6719	7096
Sex (Male)	−1.04	0.27	−1.56	−0.52	1	8451	7845
Aggression (Given)	1.11	0.19	0.74	1.48	1	8315	7884
Age	−0.05	0.23	−0.49	0.39	1	8152	7374
Grooming (Given)	0.77	0.19	0.40	1.14	1	8766	7986
Count of kin giving SHP	1.12	0.21	0.70	1.53	1	7491	8456
Count of kin not giving SHP	−0.25	0.29	−0.81	0.32	1	8159	8277
	**Family Specific Parameters:**						
Shape	0.07	0.04	0.03	0.16	1	2686	4468

SHP Received							

	Est.	Error	l-95% CI	u-95% CI	Rhat	Bulk_ESS	Tail_ESS

	**Group-Level Effects:**						
~Group (Levels = 7) sd(Intercept)	0.79	0.33	0.39	1.63	1	3673	3682
~ID (Levels = 627) sd(Intercept)	0.25	0.14	0.02	0.53	1	1268	3013
	**Population-Level Effects:**						
Intercept	−4.08	0.33	−4.78	−3.46	1	3000	3309
Sex (Male)	−0.79	0.13	−1.05	−0.53	1	7413	7753
Rank	0.79	0.13	0.55	1.04	1	7126	7931
Age	0.46	0.12	0.23	0.70	1	7722	7055
Grooming (Received)	0.67	0.13	0.42	0.93	1	7153	7918
Count of kin giving SHP	0.61	0.12	0.37	0.86	1	8291	8508
Count of kin not giving SHP	−0.54	0.15	−0.85	−0.24	1	7491	7513
	**Family Specific Parameters:**						
Shape	1.43	0.32	1.00	2.21	1	2476	3736
Zero-inflation	0.03	0.02	0.00	0.09	1	6735	6889

**Table 4.** Model estimates for rates of AHP given.

AHP Given							

	Est.	Error	l-95% CI	u-95% CI	Rhat	Bulk_ESS	Tail_ESS

	**Group-Level Effects:**						
~Group (Levels = 7) sd(Intercept)	0.57	0.30	0.17	1.34	1	3828	4233
~ID (Levels = 627) sd(Intercept)	1.19	0.16	0.86	1.48	1	2495	3652
	**Population-Level Effects:**						
Intercept	−5.68	0.31	−6.31	−5.10	1	4308	5628
SHP (Given)	3.56	0.63	2.34	4.78	1	7592	8027
Sex (Male)	−0.49	0.24	−0.96	−0.02	1	7883	8244
Aggression (Given)	2.19	0.19	1.82	2.58	1	7486	8239
Age	−0.17	0.20	−0.56	0.21	1	8164	8429
Grooming (Given)	0.07	0.20	−0.32	0.45	1	8073	7715
Count of SHP kin	0.33	0.21	−0.06	0.74	1	7725	7672
Count of non-SHP kin	0.08	0.26	−0.44	0.60	1	7031	8331
	**Family Specific Parameters:**						
Shape	0.02	0.01	0.01	0.05	1	2466	3796

**Table 5.** Models’ estimates for alopecia (top) and body condition scores (bottom).

Alopecia Ratings							

	Est.	Error	l-95% CI	u-95% CI	Rhat	Bulk_ESS	Tail_ESS

	**Smooth Terms:**						
sds(Month #)	0.51	0.20	0.24	1.00	1	3526	5116
	**Group-Level Effects:**						
~Group (Levels = 6) sd(Intercept)	2.53	1.14	1.09	5.45	1	3682	5318
sd(disc_Intercept)	0.29	0.15	0.12	0.69	1	5994	7319
~ID (Levels = 507) sd(Intercept)	2.97	0.55	2.03	4.23	1	2954	4161
~Observer (Levels = 7) sd(disc_Intercept)	0.44	0.22	0.15	1.01	1	4522	6324
	**Population-Level Effects:**						
Intercept [1]	−1.26	1.25	−4.41	0.61	1	3607	4237
Intercept [2]	4.03	1.42	0.96	6.78	1	3726	5553
Intercept [3]	7.87	1.88	4.34	11.93	1	3603	4916
Intercept [4]	11.75	2.49	7.44	17.26	1	3512	4527
Age	0.09	0.07	−0.05	0.24	1	2399	4063
Sex/Status (F/Preg.)	−0.73	0.19	−1.15	−0.42	1	4159	5392
Sex/Status (M/NA)	0.06	0.30	−0.54	0.65	1	2370	4251
Count of kin giving SHP	0.29	0.14	0.03	0.58	1	2185	3492
SHP Received? Yes.	0.55	0.15	0.30	0.89	1	4641	6172
Rank	0.64	0.19	0.32	1.06	1	2188	3667

Body Ratings							

	Est.	Error	l-95% CI	u-95% CI	Rhat	Bulk_ESS	Tail_ESS

	**Smooth Terms:**						
sds(Month #)	0.22	0.10	0.10	0.46	1	2918	4424
	**Group-Level Effects:**						
~Group (Levels = 6) sd(Intercept)	0.84	0.48	0.22	2.05	1	1465	1651
sd(disc_Intercept)	0.31	0.23	0.05	0.90	1	3241	4296
~ID (Levels = 506) sd(Intercept)	3.03	0.60	1.94	4.27	1	2130	3494
~Observer (Levels = 6) sd(disc_Intercept)	0.70	0.35	0.27	1.58	1	3466	5696
	**Population-Level Effects:**						
Intercept [1]	−10.88	2.21	−15.53	−6.86	1	2332	3340
Intercept [2]	−3.20	0.73	−4.76	−1.88	1	2626	4830
Intercept [3]	2.27	0.62	1.17	3.59	1	2719	5084
Intercept [4]	5.88	1.23	3.65	8.44	1	2262	3761
Age	2.36	0.49	1.48	3.40	1	2267	3545
Sex/Status (F/Preg.)	0.28	0.12	0.07	0.55	1	5000	6071
Sex/Status (M/NA)	−1.84	0.42	−2.73	−1.10	1	1908	3776
Count of kin giving SHP	−0.30	0.34	−1.00	0.34	1	1362	2971
SHP Received? Yes.	−0.01	0.08	−0.17	0.16	1	6773	7420
SHP Given? Yes.	−0.08	0.10	−0.28	0.11	1	6381	7304
Rank	−0.39	0.17	−0.75	−0.09	1	1217	2136

**Table 6.** Model estimates for hair cortisol concentrations.

Hair Cortisol							

	Est.	Error	l-95% CI	u-95% CI	Rhat	Bulk_ESS	Tail_ESS

	**Group-Level Effects:**						
~Group (Levels = 6) sd(Intercept)	0.07	0.04	0.03	0.17	1	4379	6232
~ID (Levels = 521) sd(Intercept)	0.17	0.01	0.15	0.18	1	5293	7700
	**Population-Level Effects:**						
Intercept	4.04	0.03	3.97	4.10	1	5172	5595
SHP (Given)	0.04	0.02	0.00	0.07	1	7848	8510
Sex (Male)	−0.02	0.02	−0.06	0.03	1	6449	8155
Aggression (Received)	−0.03	0.02	−0.06	0.01	1	7618	9161
Aggression (Given)	−0.04	0.02	−0.07	−0.01	1	7282	8856
Temperature (Max)	−0.23	0.01	−0.26	−0.21	1	8688	8372
Age	0.03	0.02	−0.01	0.06	1	6195	8506
SHP : Sex	−0.10	0.05	−0.21	0.00	1	7949	8684
	**Family Specific Parameters:**						
Sigma	0.20	0.00	0.19	0.21	1	6670	8667

## Data Availability

Files containing the data and analyses necessary to replicate these results are available in the Dryad data repository: https://doi.org/10.5061/dryad.m37pvmdbr.
