# Supplementary material for: Social Hair Plucking’s Associations with Other Behaviors and Its Health Considerations, in Captive Rhesus Macaques (Macaca mulatta)
Source: J Appl Anim Welf Sci. Author manuscript; Available in PMC 2026 Sep 16. (PMC13580905; doi:10.1080/10888705.2026.2692473)
Supplement: Supplementary file 1 [file NIHMS2197350-supplement-Supp_1.pdf]

Supplementary Materials:
Social Hair Plucking's Associations with Other Behaviors and its Health Considerations, in Captive Rhesus Macaques (*Macaca mulatta*)

Alexander J. Pritchard[1,2]*, Julia A. Salamango[1,3], Brenda McCowan[1,2]

[1] National Biomedical Research Institute, University of California, Davis, Davis, CA, USA

[2] Department of Population Health & Reproduction, School of Veterinary Medicine, University of California, Davis, Davis, CA, USA

[3] Department of Anthropology, University of California, Santa Barbara, Santa Barbara, CA, USA

*Corresponding author
E-mail: ajpritchard@ucdavis.edu (AJP)

Article DOI: http://dx.doi.org/10.1080/10888705.2026.2692473

S1. Notes on Model Selection

Model selection was performed using *loo_compare()* in brms. We selected models with clear separation from other candidate models (> 2 * se_diff from the elpd of the best performing model) or that were more biologically appropriate based on our knowledge of the system. For SHP given and received, we did not include all behaviors given and received; instead, we only included behaviors that were directionally similar. That is, for SHP given, we examined grooming and aggression given, not received.

For SHP given and received, as well as AHP given, candidate models were executed with two chains, 1500 iterations, with a warmup of 500. For the alopecia and body condition scores, candidate models were executed with two chains, 800 iterations, with a warmup of 200. As these models were more computationally intensive, we used threading to improve execution of the candidate models, but not the full model. For hair cortisol concentration, candidate models were executed with two chains, 2000 iterations, with a warmup of 500, and a thin of two.

For SHP given (**S2**), inclusion of fixed effects improved model performance relative to the random effects' models. One model was similar to the selected model and omitted the count of kin that did not SHP. We retained this effect, however, as matrilineal size might be expected to be related to access to social partners; this variable aids in understanding that count of kin is unlikely to be driving these dynamics.

For SHP received (**S3**), inclusion of fixed effects improved model performance relative to the random effects' models. All fixed effects models performed similarly to the selected model. We retained: the count of non-SHP kin, for the same rationale as with SHP given; the count of SHP kin, rather than matriline as a random effect; rank, rather than aggression received.

For AHP given (**S4**), inclusion of fixed effects improved model performance relative to the random effects' models. All fixed effects models performed similarly to the selected model. We retained: the count of non-SHP kin, for the same rationale as with SHP given; the count of SHP kin, rather than matriline size as a fixed effect; aggression given, rather than rank.

For alopecia scores (**S5, A**), inclusion of fixed effects improved model performance relative to the random effects' models. All fixed effects models performed similarly to the selected model. We retained: age as a biologically salient variable; we retained SHP received and the count of SHP kin. We had included SHP given as a variable during model selection, but did not have a strong biological rationale to retain it. The preliminary model did not show strong evidence for a

meaningful association. Observers and groups were added as random effects on the discrimination parameter after model selection. Observers may be expected to differ in their cut-offs for each score. Similarly, we expected that individuals were more likely to be judged relative to others in the same group.

For body condition scores (**S5, B**), inclusion of fixed effects improved model performance relative to the random effects' models. All fixed effects models performed similarly to the selected model. We retained: age as a biologically salient variable; we retained SHP received and the count of SHP kin; SHP given; and month as a cyclic spline, which is more logical than unlinked categorical factors. As with the alopecia model and under the same underlying rationale: observers and groups were added as random effects on the discrimination parameter.

For hair cortisol (**S6**), prior work (Pritchard et al. *in review*) had shown that inclusion of temperature, sex, and age improved model performance relative to random effects models; we did not formally reexamine this. All fixed effects models performed similarly to the selected model. We have no strong rationale for excluding other models, except via selecting the top-performing model. Due to the known effects of rank and social support, numerous hypotheses can be derived as to the action of grooming and aggression. Among rhesus macaques, the action of SHP in this context (if any) is relatively unknown. We note that there are more candidate models here as we, stepwise, compared the inclusion of SHP given and received, followed by interactions with sex (given our findings with SHP given and received), then aggression given and received, and, finally, grooming given and received. SHP given, in all models with a sex interaction, retained a similar estimate (0.03 to 0.04) with similar CIs (L-95% = -0.00 to 0.01; U-95% = 0.06 to 0.07), cumulatively providing equivocal support.

S2. Model selection comparisons for SHP Given. Selected model is bolded. Similar models based on *se_diff* and *elpd_diff*, are italicized. Order of construction is the sequence of code-writing. Order of comparison is the group at which models were first compared.

Order of		Family	Effects			Loo Comparisons	
Construction	Comparison		Offset	Random	Fixed	elpd_diff	se_diff
**15**	**4**	**Negative Binomial**	Affiliation Days	**Groups, ID**	**Sex, Aggression (Given), Age, Groom (Given), SHP Kin, Non-SHP Kin**	**0**	**0**
14	*4*	*Negative Binomial*		*Groups, ID*	*Sex, Aggression (Given), Age, Groom (Given), SHP Kin*	*-0.44*	*2.49*
18	4	Negative Binomial		Groups, ID	Sex, Aggression (Given), Age, Groom (Given), Matriline Size	-25.20	4.52
12	3	Negative Binomial		Groups, ID, Matriline	Sex, Age, Groom (Given), Aggression (Given), Matriline Size	-26.06	4.63
17	4	Negative Binomial		Groups, ID	Sex, Aggression (Given), Age, Groom (Given), Non-SHP Kin	-30.96	5.35
16	4	Negative Binomial		Groups, ID, Matriline	Sex, Aggression (Given), Age, Groom (Given)	-31.13	5.13
13	3	Negative Binomial		Groups, ID, Matriline	Sex, Rank, Age, Groom (Given), Matriline Size	-40.89	5.83
9	2	Negative Binomial		Groups, ID	(Intercept Only)	-78.73	8.86
10	2	Negative Binomial		Groups, ID, Matriline	(Intercept Only)	-80.64	8.55
5	1	Hurdle Negative Binomial		Groups, ID	(Intercept Only)	-86.15	9.24
3	1	Zero-inflated Negative Binomial		Groups, ID	(Intercept Only)	-89.21	9.19
11	2	Negative Binomial		ID, Matri-line	(Intercept Only)	-90.76	9.02
8	2	Negative Binomial		ID	(Intercept Only)	-110.30	10.10
7	2	Negative Binomial		Matriline	(Intercept Only)	-167.60	15.50
4	1	Negative Binomial		Groups, ID	(Intercept Only)	-170.48	15.37
6	2	Negative Binomial		Groups	(Intercept Only)	-183.87	18.07
2	1	Hurdle Negative Binomial	–	–	(Intercept Only)	-188.15	15.71
1	1	Zero-inflated Negative Binomial		–	(Intercept Only)	-196.25	16.39

S3. Model selection comparisons for SHP Received. Selected model is bolded. Similar models based on *se_diff* and *elpd_diff*, are italicized. Order of construction is the sequence of code-writing. Order of comparison is the group at which models were first compared.

Order of		Family	Effects			Loo Comparisons	
Construction	Comparison		Offset	Random	Fixed	elpd_diff	se_diff
**17**	**5**	**Zero-inflated Negative Binomial**		**Groups, ID**	**Sex, Rank, Age, Groom (Received), SHP Kin, Non-SHP Kin**	**0.00**	**0.00**
13	*3*	*Negative Binomial*		*Groups, ID*	*Sex, Rank, Age, Groom (Received), SHP Kin, Non-SHP Kin*	*-2.53*	*2.69*
12	*3*	*Negative Binomial*		*Groups, ID*	*Sex, Age, Aggression (Received), Groom (Received), SHP Kin, Non-SHP Kin*	*-3.10*	*6.90*
15	*4*	*Negative Binomial*		*Groups, ID*	*Sex, Rank, Age, Groom (Received), SHP Kin*	*-7.35*	*4.20*
16	*4*	*Negative Binomial*		*Groups, ID, Matriline*	*Sex, Rank, Age, Groom (Received)*	*-8.87*	*5.66*
14	*4*	*Negative Binomial*		*Groups, ID*	*Sex, Rank, Age, Groom (Received), Non-SHP Kin*	*-9.98*	*5.61*
9	2	Negative Binomial	Affiliation Days	Groups, ID, Matriline	(Intercept Only)	-59.07	11.42
6	2	Negative Binomial		Groups	(Intercept Only)	-60.80	11.80
5	1	Negative Binomial		Groups, ID	(Intercept Only)	-61.39	11.42
3	1	Zero-inflated Negative Binomial		Groups, ID	(Intercept Only)	-61.51	11.18
11	2	Negative Binomial		Groups, ID	(Intercept Only)	-61.61	11.53
10	2	Negative Binomial		ID, Matriline	(Intercept Only)	-83.39	13.17
7	2	Negative Binomial		Matriline	(Intercept Only)	-83.58	13.18
4	1	Hurdle Negative Binomial		Groups, ID	(Intercept Only)	-104.57	13.44
8	2	Negative Binomial		ID	(Intercept Only)	-120.84	14.46
2	1	Hurdle Negative Binomial	–	–	(Intercept Only)	-127.66	13.88
1	1	Zero-inflated Negative Binomial		–	(Intercept Only)	-129.31	14.08

S4. Model selection comparisons for AHP Given. Selected model is bolded. Similar models based on *se_diff* and *elpd_diff*, are italicized. Order of construction is the sequence of code-writing. Order of comparison is the group at which models were first compared.

Order of		Family	Effects			Loo Comparisons	
Construction	Comparison		Offset	Random	Fixed	elpd_diff	se_diff
**18**	**5**	**Negative Binomial**		**Groups, ID**	**SHP (Given), Sex, Aggression (Given), Age, Groom (Given), SHP Kin, Non-SHP Kin**	**0.00**	**0.00**
20	*5*	Negative Binomial		*Groups, ID*	*SHP (Given), SHP (Received), Sex, Aggression (Given), Age, Groom (Given), SHP Kin, Non-SHP Kin*	*-0.70*	*2.06*
17	*4*	Negative Binomial		*Groups, ID*	*Sex, Aggression (Given), Age, Groom (Given), Matriline Size*	*-7.61*	*6.74*
14	*4*	Negative Binomial		*Groups, ID*	*Sex, Aggression (Given), Age, Groom (Given), SHP Kin*	*-7.89*	*6.71*
13	*3*	Negative Binomial		*Groups, ID*	*Sex, Rank, Age, Groom (Given), Matriline Size*	*-7.89*	*11.80*
15	*4*	Negative Binomial		*Groups, ID*	*Sex, Aggression (Given), Age, Groom (Given), SHP Kin, Non-SHP Kin*	*-9.23*	*6.51*
19	*5*	Negative Binomial		*Groups, ID*	*SHP (Received), Sex, Aggression (Given), Age, Groom (Given), SHP Kin, Non-SHP Kin*	*-10.17*	*6.35*
12	*3*	Negative Binomial		*Groups, ID*	*Sex, Age, Groom (Given), Aggression (Given), Matriline Size*	*-11.48*	*7.06*
16	*4*	Negative Binomial		*Groups, ID*	*Sex, Aggression (Given), Age, Groom (Given), Non-SHP Kin*	*-12.00*	*7.25*
9	2	Negative Binomial	Conflict Days	Groups, ID, Matriline	(Intercept Only)	-44.73	12.94
10	2	Negative Binomial		ID, Matriline	(Intercept Only)	-45.84	12.95
5	1	Negative Binomial		Groups, ID	(Intercept Only)	-75.54	11.21
11	2	Negative Binomial		Groups, ID	(Intercept Only)	-77.59	11.01
7	2	Negative Binomial		Matriline	(Intercept Only)	-79.58	15.33
3	1	Zero-inflated Negative Binomial		Groups, ID	(Intercept Only)	-81.83	11.24
8	2	Negative Binomial		ID	(Intercept Only)	-83.67	11.39
4	1	Hurdle Negative Binomial		Groups, ID	(Intercept Only)	-120.65	14.06
2	1	Hurdle Negative Binomial		-	(Intercept Only)	-139.06	16.02
6	2	Negative Binomial		Groups	(Intercept Only)	-140.11	16.26
1	1	Zero-inflated Negative Binomial		-	(Intercept Only)	-146.05	17.03

S5. Model selection for A) Alopecia & B) Body Condition. Selected model is bolded. Similar models based on *se_diff* and *elpd_diff*, are italicized. Order of construction is the sequence of code-writing. Order of comparison is the group models were first compared in.

Order of		Family	Effects		Loo Comparisons	
Construction	Comparison		Random	Fixed	elpd_diff	se_diff
			A) Alopecia Response			
6	*1*	*Cumulative (logit)*	*Groups, ID*	*Age, Sex/Repro. Status, SHP (Given; 0/1), SHP Kin, SHP (Received; 0/1), Rank, Month (Cyclic Spline)*	*0.00*	*0.00*
3	*1*	*Cumulative (logit)*	*Groups, ID*	*Sex/Repro. Status, SHP Kin, SHP (Received; 0/1), Rank, Month (Cyclic Spline)*	*-0.12*	*1.19*
**4**	**1**	**Cumulative (logit)**	**Groups, ID**	**Age, Sex/Repro. Status, SHP Kin, SHP (Received; 0/1), Rank, Month (Cyclic Spline)**	**-0.18**	**1.19**
5	*1*	*Cumulative (logit)*	*Groups, ID*	*Age, Sex/Repro. Status, Rank, Month (Cyclic Spline)*	*-7.05*	*5.14*
2	1	Cumulative (logit)	Groups, ID	(Intercept Only)	-732.71	38.63
1	1	Cumulative (logit)	-	(Intercept Only)	-3654.13	73.26
			B) Body Condition Response			
**11**	**2**	**Cumulative (logit)**	**Groups, ID**	**Age, Sex/Repro. Status, SHP Kin, SHP (Received; 0/1), SHP (Given; 0/1), Rank, Month (Cyclic Spline)**	**0.00**	**0.00**
10	*2*	*Cumulative (logit)*	*Groups, ID*	*Sex/Repro. Status, Rank, Month (Cyclic Spline)*	*-1.94*	*3.34*
4	*2*	*Cumulative (logit)*	*Groups, ID*	*Sex/Repro. Status, SHP Kin, SHP (Received; 0/1), Rank, Month (Factor)*	*-2.75*	*4.82*
8	*2*	*Cumulative (logit)*	*Groups, ID*	*Sex/Repro. Status, SHP (Received; 0/1), Rank, Month (Cyclic Spline)*	*-3.08*	*3.24*
9	*2*	*Cumulative (logit)*	*Groups, ID*	*Sex/Repro. Status, SHP Kin, Rank, Month (Cyclic Spline)*	*-5.68*	*3.64*
7	*2*	*Cumulative (logit)*	*Groups, ID*	*Sex/Repro. Status, SHP (Received; 0/1), SHP (Given; 0/1), Rank, Month (Cyclic Spline)*	*-5.97*	*3.59*
5	*2*	*Cumulative (logit)*	*Groups, ID*	*Sex/Repro. Status, SHP Kin, SHP (Received; 0/1), Rank, Month (Cyclic Spline)*	*-7.56*	*3.48*
6	*2*	*Cumulative (logit)*	*Groups, ID*	*Sex/Repro. Status, SHP Kin, SHP (Received; 0/1), SHP (Given; 0/1), Rank, Month (Cyclic Spline)*	*-7.84*	*4.03*
3	2	Cumulative (logit)	Groups, ID	(Intercept Only)	-51.07	11.46
1	1	Cumulative (logit)	-	(Intercept Only)	-5035.24	74.39
2	1	Cumulative (probit)	-	(Intercept Only)	-5035.62	74.42

S6. Model selection comparisons for hair cortisol. Selected model is bolded. Similar models based on *se_diff* and *elpd_diff*, are italicized. Order of construction is the sequence of code-writing. Order of comparison is the group at which models were first compared.

Order of		Family	Effects		Loo Comparisons	
Construction	Comparison		Random	Fixed	elpd_diff	se_diff
**11**	**3**	Lognormal	Groups, ID	**SHP (Given) * Sex, Aggression (Received), Aggression (Given), Temperature, Age**	**0.00**	**0.00**
9	*3*			*SHP (Given) * Sex, Aggression (Given), Temperature, Age*	*-1.01*	*2.39*
15	*5*			*SHP (Given) * Sex, Aggression (Given), Temperature*	*-2.07*	*2.72*
13	*4*			*SHP (Given) * Sex, Aggression (Given) , Temperature, Aggression (Received), Groom (Received), Age*	*-2.42*	*1.97*
14	*4*			*SHP (Given) * Sex, AHP (Given), Aggression (Given), Aggression (Received), Groom (Given), Groom (Received), Temperature, Age*	*-2.75*	*2.05*
5	*2*			*SHP (Received), SHP (Received):Sex, SHP (Given) * Sex, Temperature, Age*	*-2.91*	*5.56*
12	*4*			*SHP (Given) * Sex, Aggression (Given), Aggression (Received), Groom (Given), Groom (Received), Temperature, Age*	*-3.18*	*1.93*
11	4			SHP (Given) * Sex, Aggression (Received), Aggression (Given), Groom (Given), Temperature, Age	-4.04	1.78
10	*3*			*SHP (Given) * Sex, Aggression (Received), Temperature, Age*	*-5.19*	*2.77*
1	*1*			*Sex, Temperature, Age*	*-5.46*	*4.68*
6	*2*			*SHP (Received), SHP (Given) * Sex, Temperature, Age*	*-5.95*	*4.72*
3	*1*			*SHP (Received), Sex, Temperature, Age*	*-7.29*	*4.69*
4	*1*			*SHP (Received), SHP (Given), Sex, Temperature, Age*	*-7.31*	*4.71*
7	*2*			*SHP (Received) * Sex, SHP (Given), Temperature, Age*	*-7.37*	*4.89*
8	*2*			*SHP (Given) * Sex, Temperature, Age*	*-7.51*	*5.01*
2	*1*			*SHP (Given), Sex, Temperature, Age*	*-9.38*	*5.09*

S7. Model Fit Summary

For the SHP given model, there was one divergent transition among 9000 post-warmup draws. Among pareto estimates, 14 of the observations (1.8%) had a k > 1.0; 126 observation (16.3%) had a k > 0.7. Exact cross-validation using *reloo()* for the observations with k > 1.0 indicated that these observations were not problematic with exact estimates, as opposed to the approximate estimates calculated with *loo()*. Note that the majority of subjects did not have more than one sample. Thus, removal of the ID random effect resolved all very bad pareto estimates (k > 1.0), and only 3 observations exceeded 0.7. The model outcomes were qualitatively unchanged, though the count of SHP kin was more uncertain in the model without ID (estimate was 0.74 and 95% CI ranged from 0.28 – 1.21, as opposed to the reported model's 1.12, with Cis from 0.70 – 1.53).

For the SHP received model, there were six divergent transitions among 9000 post-warmup draws. None of the observations had a k > 1.0; one observation (0.1%) had a k > 0.7. Exact cross-validation using *reloo()* for the observations with k > 1.0 indicated that these observations were not problematic with exact estimates. Similarly, removal of the ID random effect resolved the one bad estimate and the model outcomes were qualitatively unchanged: fixed effect estimates were within ±0.01 between the two models.

For the AHP given model, there were two divergent transitions among 9000 post-warmup draws. Four of the observations had a k > 1.0; 45 observations (5.8%) had a k > 0.7. Exact cross-validation using *reloo()* for the observations with k > 1.0 indicated that these observations were not problematic with exact estimates. Similarly, removal of the ID random effect resolved all bad or very bad pareto estimates. The model outcomes were qualitatively unchanged: fixed effect estimates were within ±0.13 between the two models.

For the alopecia model, there were 26 divergent transitions among 9000 post-warmup draws. None of the observations had a k > 1.0; four of the 9284 observations had a k > 0.7. Because these observations were a very slim margin of a large total sample pool (< 0.05%), they were very unlikely to be informing our major findings.

For the body condition model, there were six divergent transitions among 9000 post-warmup draws. One of the observations had a k > 1.0; 45 of the 9284 observations had a k > 0.7. Because these observations were a slim margin of a large total sample pool (0.7%), they were very unlikely to be informing our major findings.

For the hair cortisol model, there were 8 divergent transitions among 10,000 post-warmup draws. Two of the observations had a $k > 1.0$; 13 observations (0.9%) had a $k > 0.7$. Exact cross-validation using *reloo()* for the observations with $k > 1.0$ indicated that these observations were not problematic with exact estimates. Similarly, removal of the ID random effect resolved all bad or very bad pareto estimates. The model outcomes were qualitatively unchanged, though SHP given was more certain in the model without ID.

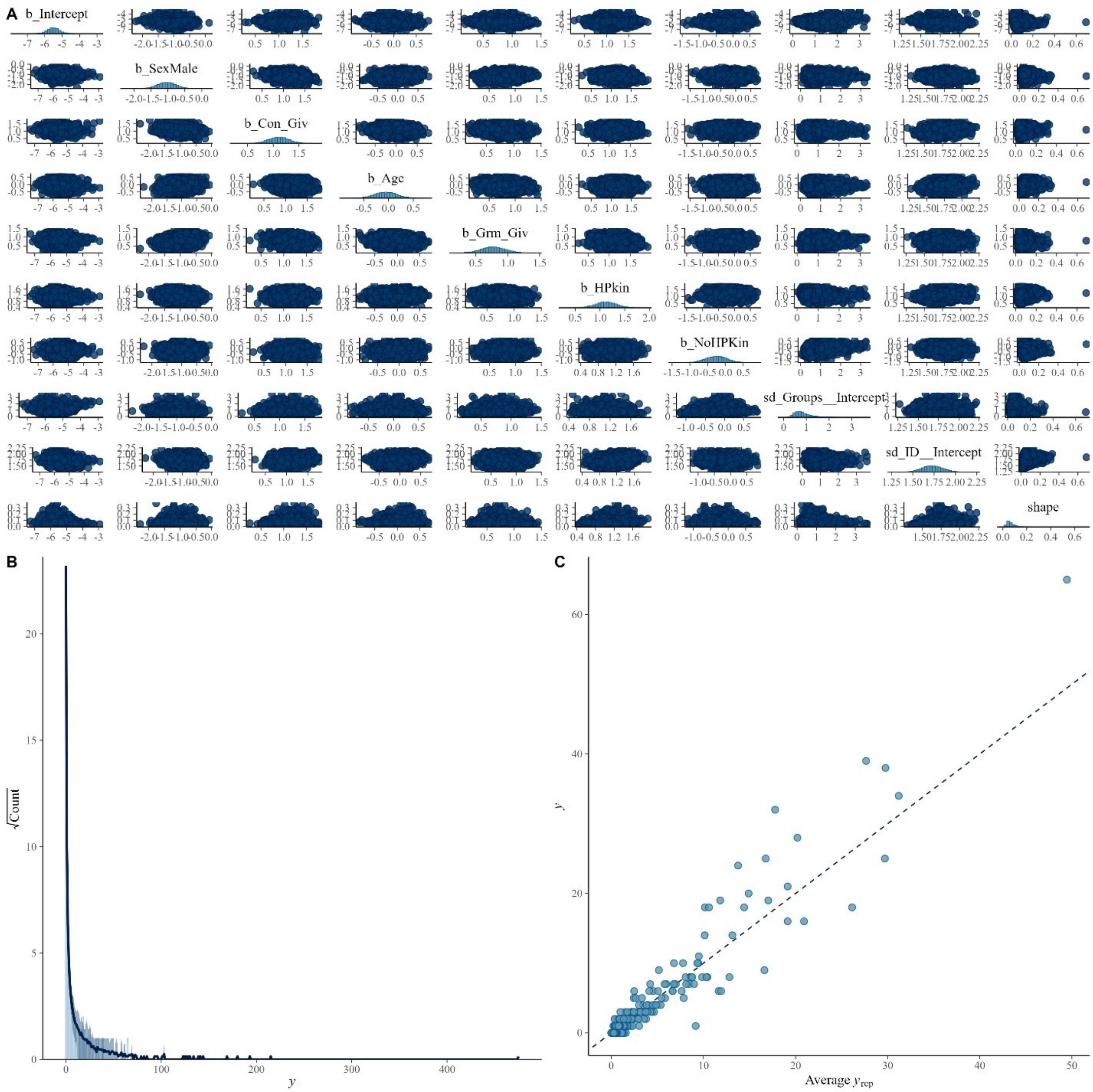

S8. Pairs plot and posterior predictive checks for models of SHP given.

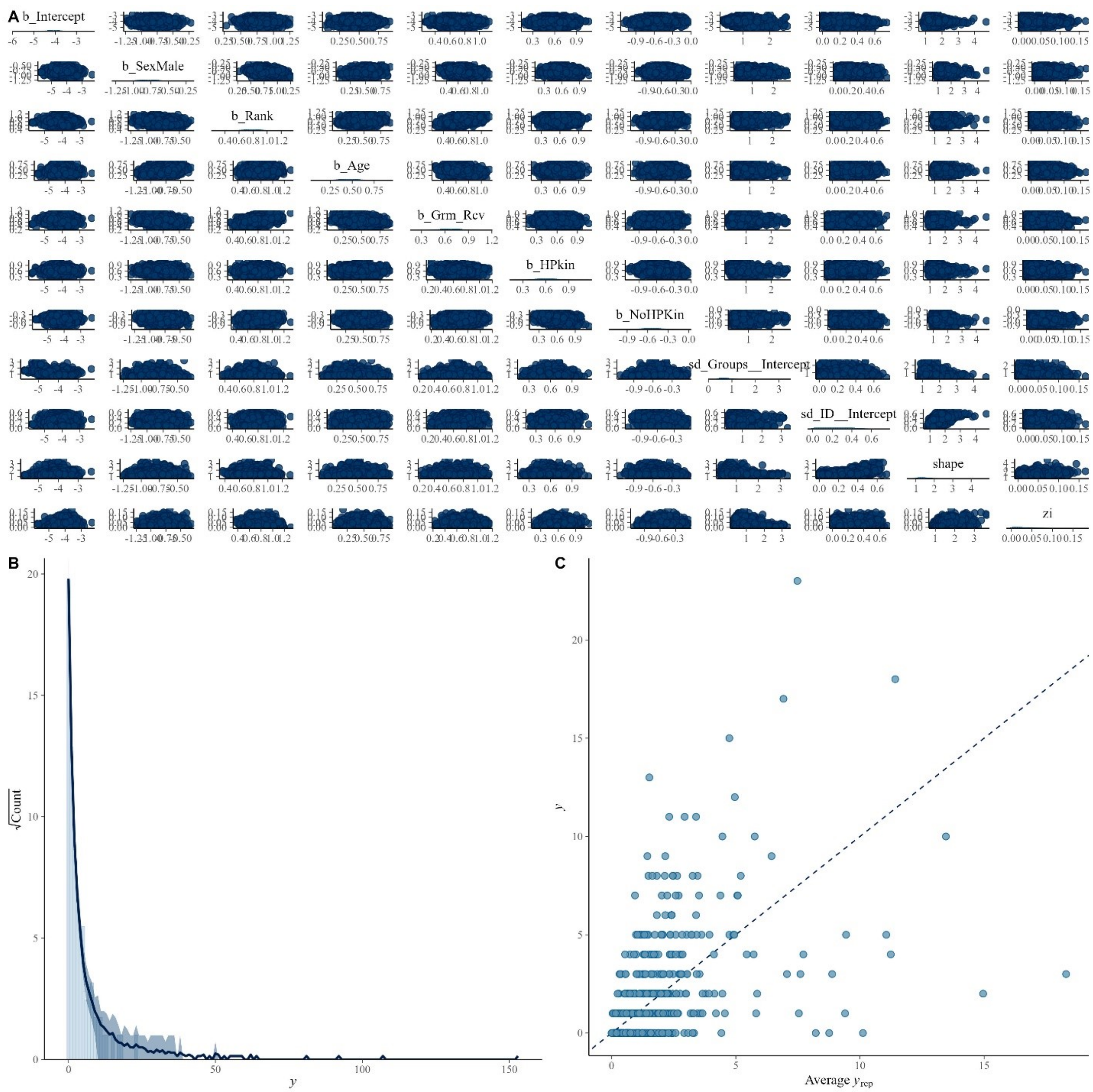

S9. Pairs plot and posterior predictive checks for models of SHP received.

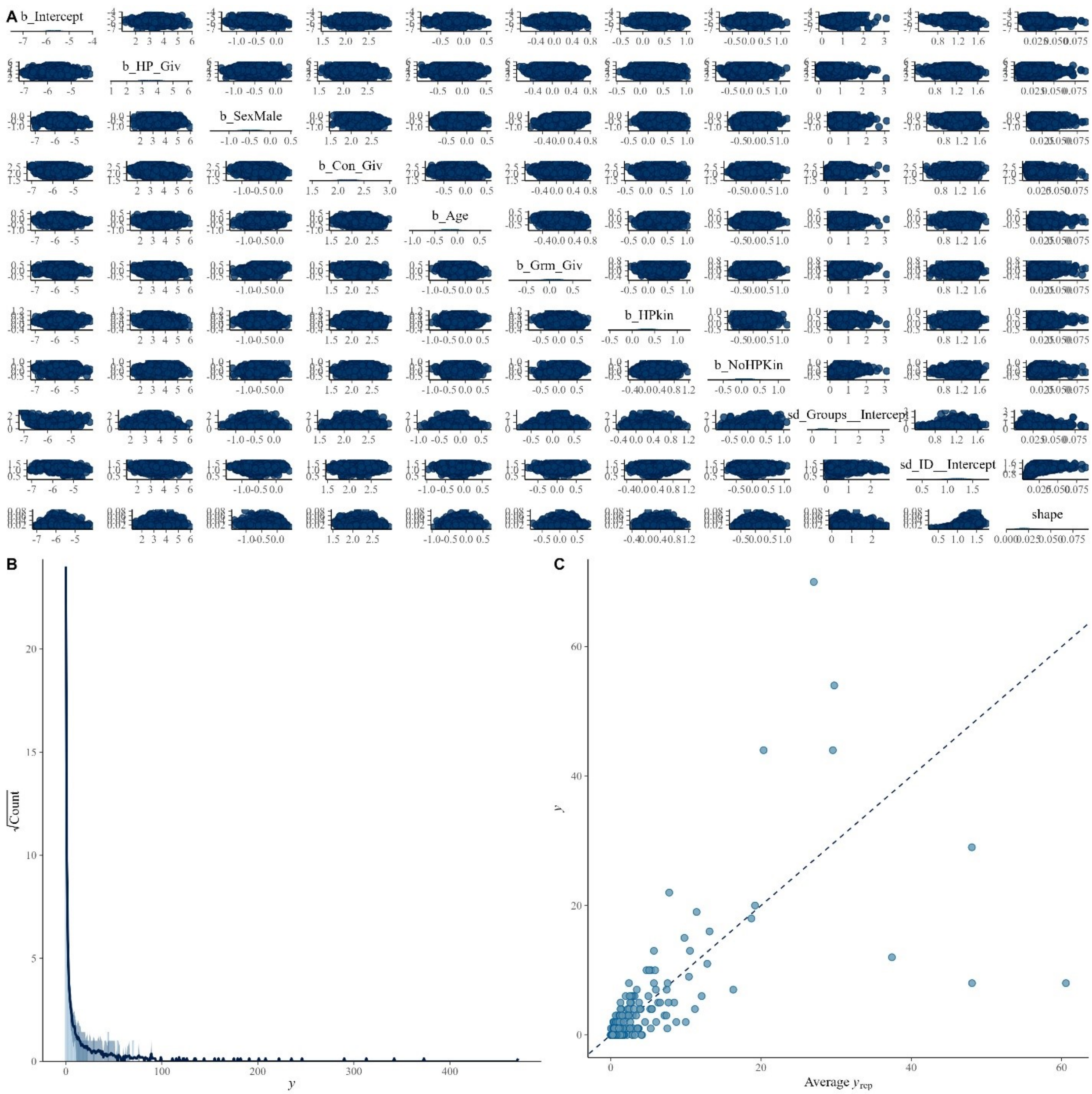

S10. Pairs plot and posterior predictive checks for models of AHP given.

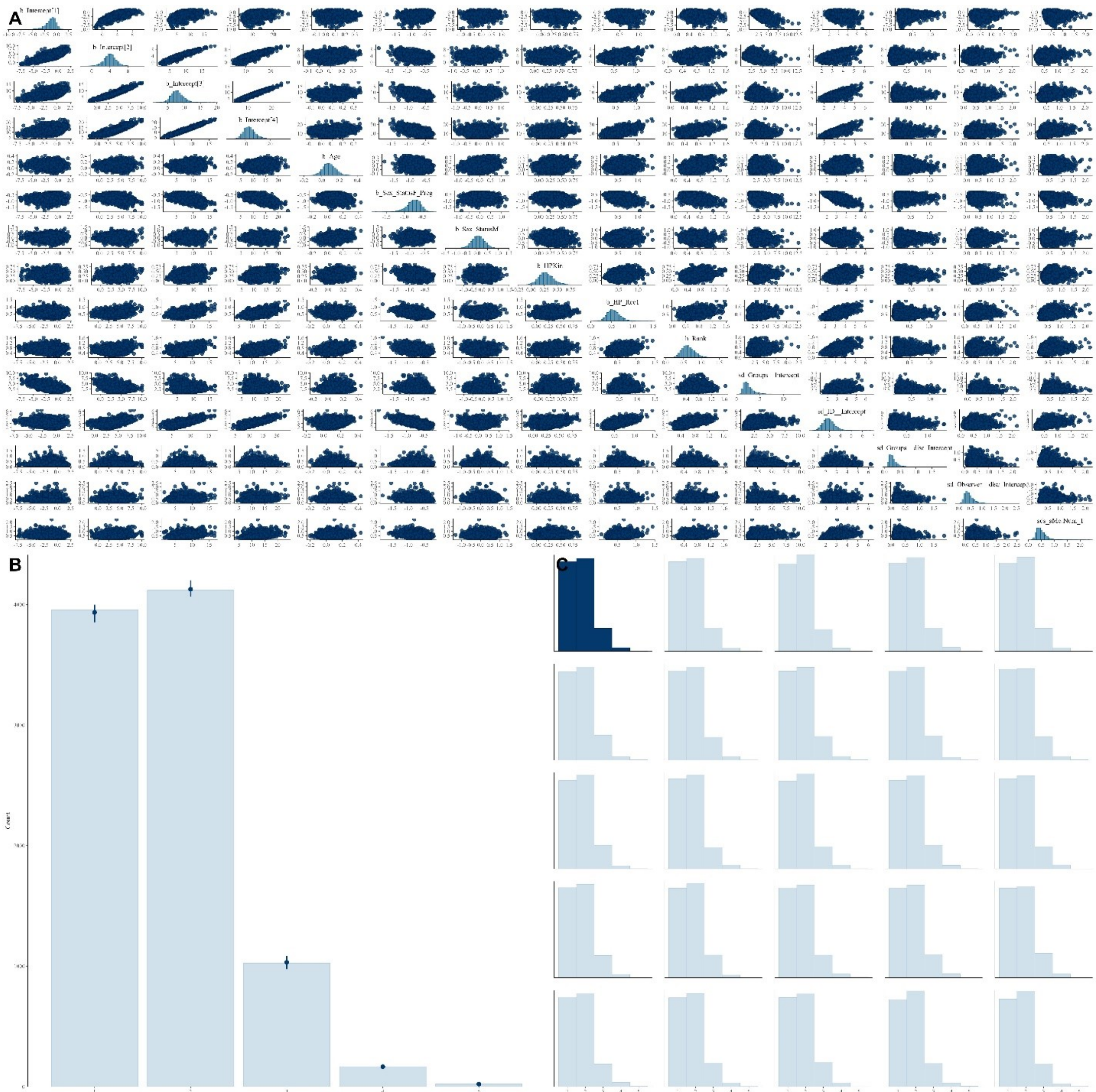

S11. Pairs plot and posterior predictive checks for models of alopecia scores.

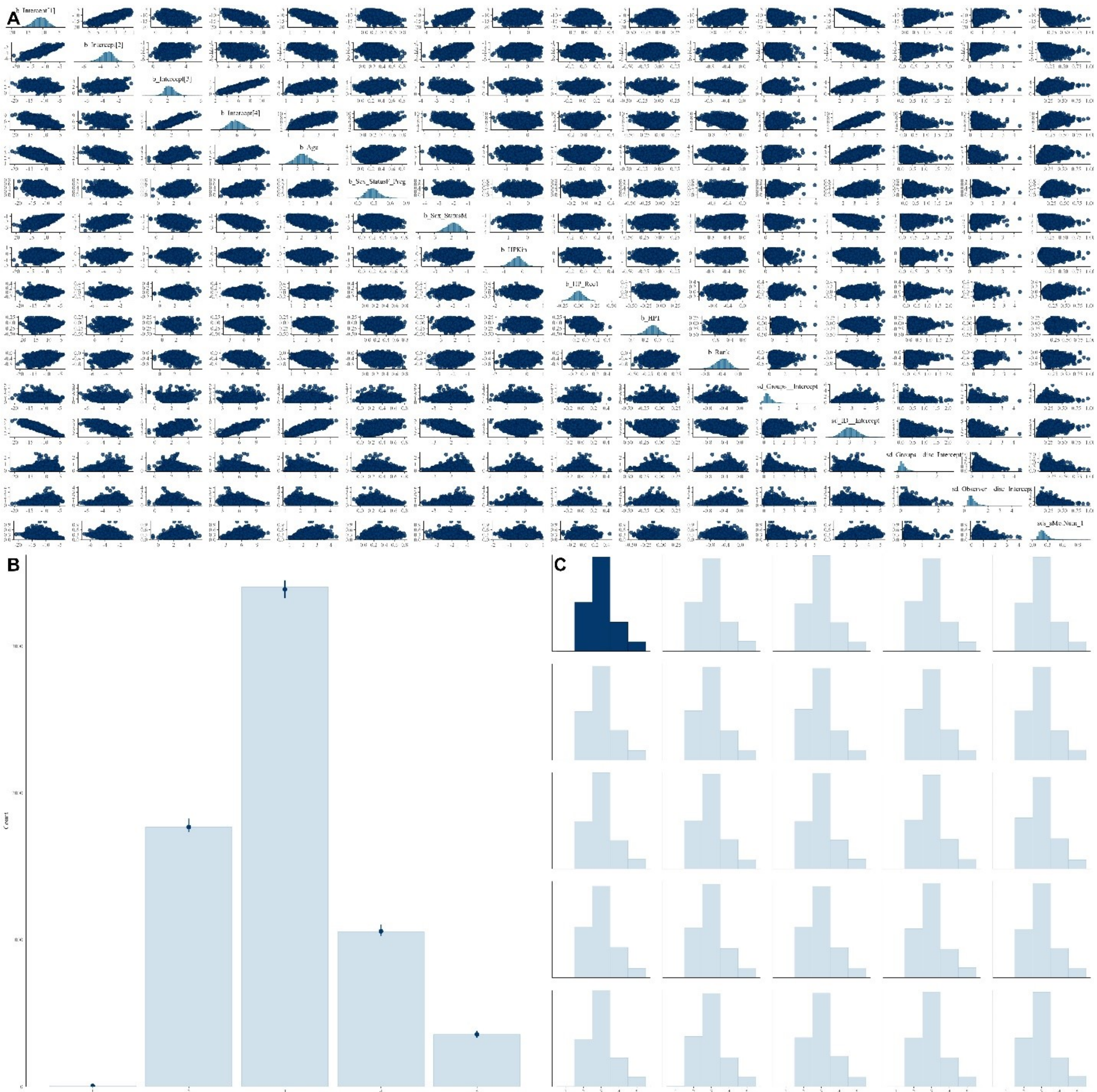

S12. Pairs plot and posterior predictive checks for models of body condition scores.

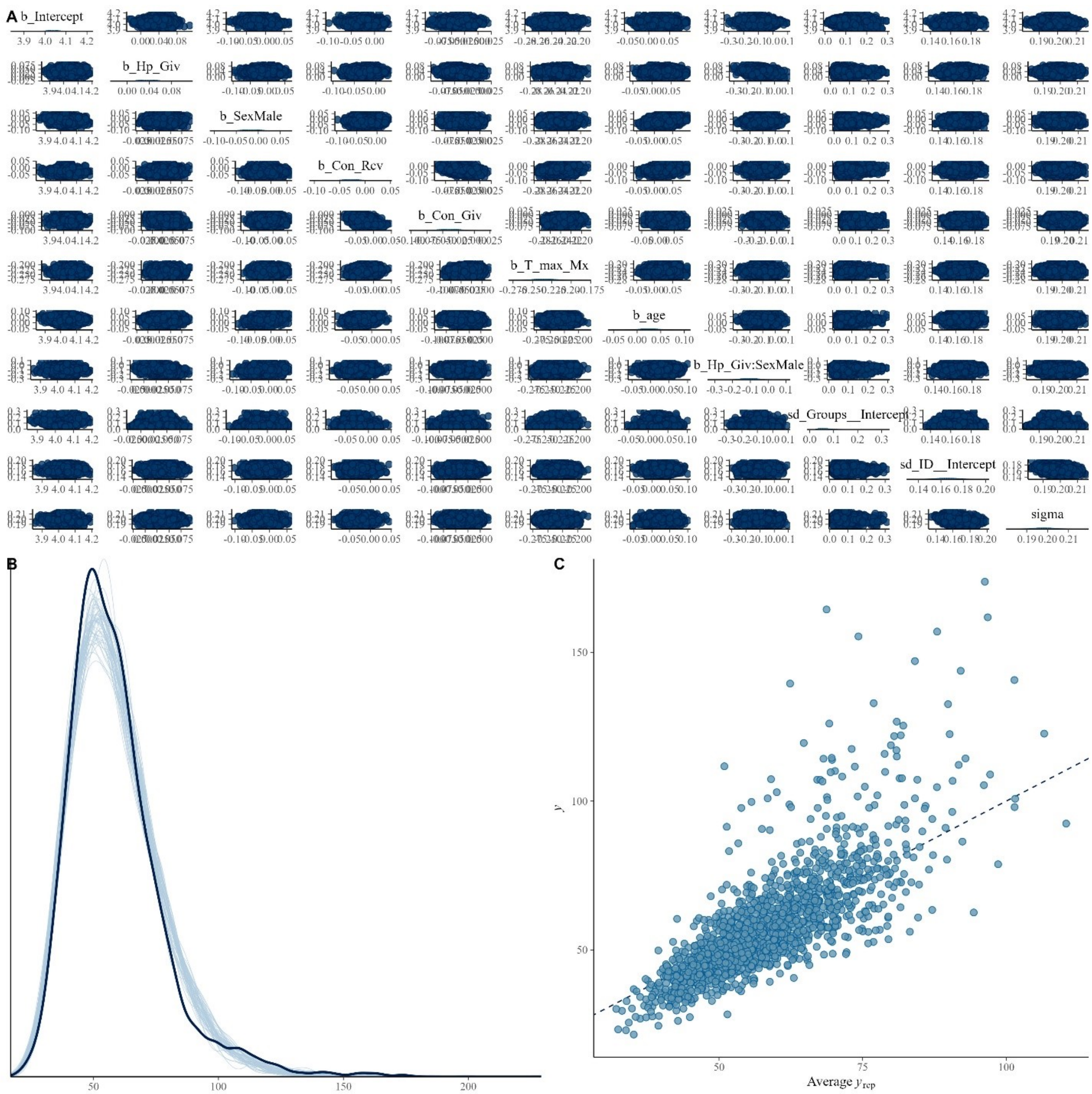

S13. Pairs plot and posterior predictive checks for models of hair cortisol concentrations.

S14. Model estimates for rates of AHP given, with 126 observations excluded.

AHP Given

	Est.	Error	l-95% CI	u-95% CI	Rhat	Bulk_ESS	Tail_ESS
**Group-Level Effects:**							
~Group (Levels = 7) sd(Intercept)	0.58	0.29	0.18	1.30	1	4467	4233
~ID (Levels = 627) sd(Intercept)	1.34	0.12	1.12	1.58	1	4494	6822
**Population-Level Effects:**							
Intercept	-5.85	0.29	-6.43	-5.29	1	5393	3750
SHP (Given)	3.55	0.59	2.38	4.72	1	7388	8317
Sex (Male)	-0.47	0.23	-0.93	-0.02	1	7132	8288
Aggression (Given)	2.12	0.18	1.77	2.48	1	7005	7653
Age	-0.14	0.19	-0.52	0.24	1	7712	8082
Grooming (Given)	0.11	0.19	-0.27	0.48	1	8242	8223
Count of SHP kin	0.32	0.20	-0.06	0.72	1	7515	7801
Count of non-SHP kin	0.08	0.26	-0.43	0.59	1	5942	3701
**Family Specific Parameters:**							
Shape	0.05	0.02	0.03	0.09	1	5027	6916

For the AHP given model with excluded observations, there were 17 divergent transitions among 9000 post-warmup draws. Cross-validations indicated that 14 observations had a $k > 1.0$. As with other models, removal of the ID random effect resolved all bad or very bad pareto estimates. The model outcomes were qualitatively unchanged with or without random effects. This model explained 64% of variation with random effects (conditional $R^2 = 0.64$, error = 0.05, L-U 95% CI: 0.54 & 0.73) and 21% without (marginal $R^2 = 0.21$, error = 0.12, L-U 95% CI: 0.04 & 0.47).
